# Photofrin Based Photodynamic Therapy and miR-99a Transfection Inhibited FGFR3 and PI3K/Akt Signaling Mechanisms to Control Growth of Human Glioblastoma *In Vitro* and *In Vivo*

**DOI:** 10.1371/journal.pone.0055652

**Published:** 2013-02-07

**Authors:** Mrinmay Chakrabarti, Naren L. Banik, Swapan K. Ray

**Affiliations:** 1 Department of Pathology, Microbiology, and Immunology, University of South Carolina School of Medicine, Columbia, South Carolina, United States of America; 2 Department of Neurosciences, Medical University of South Carolina, Charleston, South Carolina, United States of America; The University of Kansas Medical center, United States of America

## Abstract

Glioblastoma is the most common malignant brain tumor in humans. We explored the molecular mechanisms how the efficacy of photofrin based photodynamic therapy (PDT) was enhanced by miR-99a transfection in human glioblastoma cells. Our results showed almost similar uptake of photofrin after 24 h in different glioblastoma cells, but p53 wild-type cells were more sensitive to radiation and photofrin doses than p53 mutant cells. Photofrin based PDT induced apoptosis, inhibited cell invasion, prevented angiogenic network formation, and promoted DNA fragmentation and laddering in U87MG and U118MG cells harvoring p53 wild-type. Western blotting showed that photofrin based PDT was efficient to block the angiogenesis and cell survival pathways. Further, photofrin based PDT followed by miR-99a transfection dramatically increased miR-99a expression and also increased apoptosis in glioblastoma cell cultures and drastically reduced tumor growth in athymic nude mice, due to down regulation of fibroblast growth factor receptor 3 (FGFR3) and PI3K/Akt signaling mechanisms leading to inhibition of cell proliferation and induction of molecular mechanisms of apoptosis. Therefore, our results indicated that the anti-tumor effects of photofrin based PDT was strongly augmented by miR-99a overexpression and this novel combination therapeutic strategy could be used for controlling growth of human p53 wild-type glioblastomas both *in vitro* and *in vivo.*

## Introduction

Photodynamic therapy (PDT) is built on the selective accumulation of a photosensitizer (PS) in cancer cells followed by photo-induced generation of highly cytotoxic singlet oxygen and other reactive oxygen species [1], which induce oxidative stress leading to necrotic or apoptotic cell death. In general, PDT provides a new tactic for an effective treatment of the hard-to-treat tumors like glioblastoma.

Glioblastoma is the most common and most aggressive type of primary brain tumor in humans. The median survival time of glioblastoma patients is 14.6 months [2]. One of the important characteristic features of glioblastoma cells is their diffuse infiltrative nature [3]. Recently, it has been observed that fluorescence-guided resection and repetitive PDT can significantly prolong median survival in glioblastoma patients [4], [5]. Despite these promising observations, various issues have to be addressed to optimize PDT as a useful therapeutic option for glioblastoma patients.

Photofrin is an exogenous PS that readily accumulates in the cancer cells [6]. Originally, the goal of PDT in oncology was to completely eliminate localized tumors. Recently, PDT regimens have been applied to elicit vascular-targeting and also anti-tumor immune effects [7]–[10]. These new studies indicate that PDT can also be a rational treatment for the non-superficial tumors like glioblastoma.

The invasive nature of glioblastoma cells causes indistinguishable invading edges between normal and malignant brain tumor tissue [11]. Therefore, one of the main purposes of PDT is to eliminate the residual tumor cells in the margins of the resection area but at the same time minimize the damage to the surrounding normal brain tissue [12]. However, due to the limited penetration of light in tissues, tumor cells located at deeper sites beneath the resection cavity may not receive sufficient light illumination. Therefore, studying the response of photosensitized tumor cells receiving sub-lethal light doses may aid in determining the prognosis of glioblastoma patients undergoing PDT [13]–[18].

MicroRNAs (miRs) are a class of highly conserved short non-coding RNAs, which suppress protein expression through inhibiting mRNA translation or inducing mRNA degradation by binding to the 3′ untranslational region (3′UTR) of the target mRNAs [19], [20]. In addition to involvement in diverse biological processes, it has already been well demonstrated that deregulation or dysfunction of miRs can contribute to development of various cancers [21]. Recent studies show that miRs have significant roles in vascular development and angiogenesis [22]. In fact, different investigators have identified several miRs such as miR-130a [23], miR-210 [24], miR-378 [25], miR-126 [26], miR-221 [27], miR-222 [28], miR-15 [29], miR-16 [30], and miR-17–92 cluster [31], which are involved in modulating endothelial cell function and angiogenesis. Growing evidence indicates that deregulation of miRs can also contribute to development of glioblastoma by modulation of cell growth, apoptosis, migration, or invasion [32]. Overexpression of miR-124a significantly inhibited cell migration and invasion of human glioblastoma A172 cells [33]. Thus, more extensive investigations are needed to identify specific miRs, which can be used in diagnosis, prognosis, or devising therapeutic target in glioblastoma. Down regulation of miR-99a has been reported in several human cancers [34], suggesting the important role of low level of miR-99a in cancer development. Recent studies showed that miR-99 family was upregulated following DNA damage and miR-99 expression correlated well with radiation sensitivity due to down regulation of SNF2H, a miR-99 family target [35]. However, the role of miR-99a in glioblastoma development still remains unknown. Although PDT is used in clinical practice, molecular mechanisms underlying the differential radiosensitivity of some tumors to fractionated therapy are not yet entirely understood [36].

We studied the uptake and the cytotoxic effect of photofrin, inhibition of cell invasion, prevention of angiogenic network formation, and induction of DNA fragmentation in human glioblastoma cells. We also investigated the important molecular mechanisms for the anti-cancer effects of the combination of photofrin based PDT and miR-99a overexpression in human glioblastoma cells in culture and xenograft models. Photofrin based PDT could increase miR-99a expression to some extent in the p53 wild-type glioblastoma cells. Photofrin based PDT followed by transfection of miR-99a mimic could significantly increase induction of apoptosis due to dramatic miR-99a overexpression. Our results also showed that photofrin based PDT followed by miR-99a overexpression could directly or indirectly inhibit fibroblast growth factor receptor 3 (FGFR3) and PI3K/Akt signaling mechanisms to trigger the p53-mediated caspase-dependent pathway of apoptosis in the p53 wild-type glioblastoma cells both in vitro and in vivo. In essence, our current findings clearly demonstrated that the combination of photofrin based PDT and miR-99a overexpression could serve as a new therapeutic strategy for an effective treatment of human glioblastomas harvoring p53 wild-type.

## Materials and Methods

### Cell Culture and Treatments

Human glioblastoma A172 (p53 wild-type), U118MG (p53 wild-type), U87MG (p53 wild-type), LN18 (p53 mutant), and T98G (p53 mutant) cell lines were obtained from the American Type Culture Collection (ATCC, Manassas, VA, USA). SNB19 (p53 mutant) was procured from the National Cancer Institute (Frederick, MD, USA). U118MG and U87MG cells were maintained in 1×RPMI 1640 medium, T98G and A172 cells were grown in 1×DMEM medium, and SNB19 and LN18 were grown in DMEM/F12 media. All media were supplemented with 10% fetal bovine serum (FBS) and 1% penicillin and 1% streptomycin (GIBCO/BRL, Grand Island, NY, USA). All cell lines were maintained in a fully-humidified incubator containing 5% CO_2_ at 37°C. The media and FBS were purchased from Mediatech (Atlanta Biologicals, Atlanta, GA, USA). Photofrin (Axcan Scandipharm, Birmingham, AL, USA) was prepared in 5% dextrose to make 2.5 mg/ml stock solution and stored in the dark at −70°C. In all experiments, control cultures contained the same volume of vehicle that was used in the treatment with photofrin. Following the treatments and light exposure using Quantum Warp 10 (Quantum Devices, Barneveld, WI, USA), cells were used for determination of morphological and biochemical features of apoptosis, residual cell viability, flow cytometry, and alterations in expression of specific proteins. After photofrin based PDT and miR-99a transfection, the anti-cancer effects were analyzed at the cellular and molecular (DNA, RNA, and protein) levels.

### Drug Uptake Assay

All six glioblastoma cell lines were separately seeded (10^6^ cells) overnight in 35-mm culture dishes. Before addition of drugs, the cells were rinsed with **phosphate-buffered saline** (**PBS, pH 7.4**) and incubated with a defined concentration of photofrin for various periods of time in **serum**-free medium. After drug addition, adequate care was taken to avoid exposure to light. Following incubation with photofrin, cells were trypsinised and washed with **PBS, pH 7.4,** to remove any residual drug and then resuspended in 500 µl **PBS**, pH 7.4. The cellular fluorescence was quantified using a Beckman Coulter Epics® Elite flow cytometer (Beckman Coulter, Brea, CA, USA). Fluorescence intensity of photofrin in all six cell lines were measured via **PMT4** channel after passing a 610-nm long pass filter with excitation at 488 nm. Total 2×10^4^ events were recorded for each sample and the concentration of the drug in cells was expressed as the mean fluorescence intensity per cell.

### Determination of Residual Cell Viability Using the 3-(4, 5-dimethylthiazol-2-yl)-2, 5-diphenyl Tetrazolium Bromide (MTT) Assay

The residual cell viability was determined by the MTT assay at different light doses (0–2.5 J/cm^2^) and at different concentrations (0–60 µg/ml) of photofrin. Briefly, the cells were seeded (1×10^4^ cells/well) into the 96-well microculture plates and allowed to attach overnight. The medium was removed and replaced with fresh medium with or without photofrin. The cells were incubated for 4 h and then irradiated. After photofrin based PDT, the cells were washed twice with PBS, pH 7.4, and processed for the MTT assay according to the manufacturer’s protocol (Chemicon International, Temecula, CA, USA). The absorbance was determined at 570 nm using a multiwell plate reader (BioTek, Winooski, VT, USA).

### In Situ Wright Staining for Detection of Morphological Features of Apoptosis

After 4 h of photofrin (0–50 µg/ml) treatment, cells were irradiated (1 J/cm^2^) and incubated for 24 h. Then, both adherent and non-adherent cells were centrifuged at 2,500 rpm for 10 min and washed twice with PBS, pH 7.4, before being fixed in 95% (v/v) ethanol. The cells were allowed to dry before in situ Wright staining according to the manufacturer’s protocol (Thermo Fisher Scientific, Kalamazoo, MI, USA). The morphological features of the cells (*n* = 300) were observed under the light microscope. The morphological features of apoptotic cells included at least one of such characteristics as cell shrinkage, chromatin condensation, and membrane-bound apoptotic bodies.

### Annexin V-fluorescein Isothiocyanate (FITC)/Propidium Iodide (PI) Double Staining and Flow Cytometric Analysis of Apoptotic Populations

Cells were harvested at the completion of the respective drug and light treatments (*n* = 3), and washed twice with PBS, pH 7.4, before being fixed with 70% (v/v) ethanol for 15 min on ice. Subsequently, the cells were centrifuged at a low rpm to obtain pellets and residual alcohol was aspirated. Cells were then digested with DNase-free RNase A (2 mg/ml) for 30 min at 37°C. For Annexin V-FITC/PI double staining, cells were processed as per the manufacturer’s instructions (BD Bioscience, San Jose, CA, USA), and then analyzed on the Epics XL-MCL Flow Cytometer (Beckman Coulter, Fullerton, CA, USA). We considered Annexin V-FITC negative and PI positive cells (quadrant A1) as mechanically injured during the experiment; both Annexin V-FITC and PI positive cells (quadrant A2) as late necrotic; both Annexin V-FITC and PI negative cells (quadrant A3) as normal; and Annexin V-FITC positive and PI negative cells (quadrant A4) as early apoptotic. The flow cytometric results of apoptotic cells (Annexin V-FITC positive and PI negative cells in quadrant A4) from different treatments were then analyzed for statistical significance.

### In Vitro Matrigel Invasion Assay

Transwell inserts (10.5 mm diameter, 8 µm pore size) were procured (BD Biosciences, San Jose, CA, USA), coated with 3.5 mg/ml Matrigel (BD Biosciences, San Jose, CA, USA) to form a thin continuous layer, and allowed to gel overnight at 37°C. About 5×10^4^ cells in serum-free medium (with or without photofrin) were seeded into each insert (the upper chamber) and complete medium to act as a chemo attractant was placed in the lower chamber. After 4 h incubation, radiotherapy was performed and the cells were incubated for another 20 h to allow cell invasion through the Matrigel layer. The cells on the upper surface of insert membrane were then removed by wiping with a cotton swab and cells migrated to the lower surface were fixed with 10% (v/v) formaldehyde and stained with crystal violet. The number of control cells (without drug and radiation) that migrated through the Matrigel was counted and compared with the number of treated cells.

### In Vitro Angiogenic Network Formation and Expression of Vascular Endothelial Growth Factor (VEGF)

The in vitro angiogenic network formation was examined to evaluate the effect of photofrin based PDT on the angiogenic network formation ability of human microvascular endothelial (HME) cells (Cascade Biologics, Portland, OR, USA) in co-culture with a human glioblastoma cell line. Human glioblastoma U87MG and U118MG cells (10^4^ cells/well) were separately seeded into 4-well chamber slides. After 24 h, the cells were treated with different concentrations (0, 10, 20, and 50 µg/ml) of photofrin. The cells were incubated for 4 h, irradiated, and then co-cultured with HME cells (2×10^4^ cells/well) in a 50∶50 mixture of serum-free medium and HME medium (Cascade Biologics, Grand Island, NY, USA). The co-cultures were terminated after 72 h, cells were fixed in cold 95% (v/v) ethanol and treated with the von Willebrand factor VIII antibody (Santa Cruz Biotechnology, Santa Cruz, CA, USA) followed by biotinylated secondary antibody (Santa Cruz Biotechnology, Santa Cruz, CA, USA). After washings, the slides were further treated with horseradish peroxidase conjugated streptavidin. The final stain was developed using 3% (v/v) 3-amino-9-ethylcarbzole in N,N-dimethylformamide. The cells were viewed under the Olympus BX-53 microscope (Olympus America, Center Valley, PA, USA) and digitally photographed. The images were quantified for assessing angiogenic network formation using the Image-Pro Discovery software (Media Cybernetics, Silver Spring, MD, USA). We carried out another set of same angiogenic network formation assay following same treatments and incubations as described above and then performed the in situ immunofluorescence microscopy to examine and quantify the changes in expression of VEGF in glioblastoma cells in co-cultures. Briefly, the co-cultures were fixed with 4% paraformaldehyde and permeabilized with 0.1% Triton X-100 containing 2% bovine serum albumin (BSA). After washing with PBS, pH 7.4, the co-cultures were treated with (1∶100) FITC conjugated goat anti-rabbit VEGF primary IgG antibody (Santa Cruz Biotechnology, Santa Cruz, CA, USA) for 1 h at room temperature. Then, co-cultures were washed thrice with PBS, pH 7.4, and examined under the Olympus BX-53 microscope (Olympus America, Center Valley, PA, USA). Levels of expression of VEGF were quantified using the public domain ImageJ software (http://rsb.info.nih.gov/ij).

### Alkaline Comet Assay and DNA Laddering Assay

For detection of DNA fragmentation due to apoptosis, we first performed alkaline comet assay [37]. After the treatments and irradiation, the cells (1×10^5^ cells/ml) were mixed with low-temperature-melting agarose (Sigma, St. Louis, MO, USA) at a ratio of 1∶10 (v/v) and spread on the slides. The slides were submerged in pre-cooled lysis solution (2.5 M NaCl, 100 mM EDTA, 10 mM Tris-HCl, pH 10, and 1% Triton X-100) at 4°C for 1 h. After lysis and rinsing, the slides were equilibrated in 40 mM Tris/boric acid, pH 8.3, 2 mM EDTA (TBE) buffer and subjected to electrophoresis at 1.0 V/cm^2^ for 20 min in TBE buffer. Following electrophoresis, DNA was stained by immersing the slides in PI (2.5 mg/ml) solution for 1 h. Slides were briefly rinsed and placed on wet towels in air-tight and light-tight boxes and fluorescence microscopic study was performed. We then performed DNA laddering assay. To examine the DNA laddering due to apoptotic cell death [38], internucleosomal DNA fragments were extracted using a DNA isolation kit (Qiagen, Valencia, CA, USA). Then, DNA sample (10 µl) was mixed with 4 µl loading dye and analyzed by electrophoresis on 1% agarose gels (Life Technologies, Grand Island, NY, USA) pre-stained with 1 mM ethidium bromide (Sigma, St. Louis, MO, USA). Each agarose gel electrophoresis experiment included a DNA (100 bp ladder) marker (Life Technologies, Grand Island, NY, USA).

### Transfection of U87MG and U118MG Cells with miR-99a Mimic

U87MG and U118MG cells were separately seeded (5×10^5^ cells/well) in 6-well plates. Next day, the used medium was replaced by the fresh medium containing 2% FBS and photofrin (50 µg/ml). After 4 h, cells were irradiated (1 J/cm^2^) and incubated for another 4 h. Then, the cells were transfected with miR-99a oligomeric RNA at 50 nM final concentration using 20 µl Lipofectamine 2000 reagent (Invitrogen, Carlsbad, CA, USA) and Opti-MEM medium following manufacturer’s protocol (Invitrogen, Carlsbad, CA, USA). After 12 h of transfection, medium was changed and cells were incubated for another 24 h. Then, cells were analyzed by flow cytometry, Western blotting, and real-time quantitative reverse transcription-polymerase chain reaction (qRT-PCR).

### RNA Extraction and cDNA Synthesis

After each desired treatment, irradiation, and miR-99a transfection, total RNA was extracted from 3×10^6^ cells using TRIZOL according to the manufacturer’s protocol (Invitrogen, Carlsbad, CA, USA). Isolated total RNA was briefly exposed to RNase-free DNase I and 1 µg total RNA was reverse transcribed to cDNA using the gene-specific primers [39] and Thermoscript reverse transcriptase (Invitrogen, Carlsbad, CA, USA). The gene specific miR-99a antisense primer (10 µM) and U6 RNA (control) were used for the reverse transcription (RT) experiments.

### Determination of Levels of Expression of miR-99a by Real-time qRT-PCR

The expression of miR-99a precursor was determined using real-time qRT-PCR method [40] with some modifications. Briefly, master mix (3 µl) containing all of the reaction components except the primers were dispensed into a real-time qRT-PCR plate (Applied Biosystems, Carlsbad, CA, USA). The master mix contained 0.5 µl 10×PCR buffer, 0.7 µl 25 mM MgCl_2_, 0.1 µl 12.5 mM dNTPs, 0.01 µl UNG, 0.025 µl AmpliTaq Gold® DNA polymerase, 0.5 µl diluted cDNA (1∶100), and water to 3 µl. All of the PCR reagents were from the SYBR green core reagent kit (Applied Biosystems, Carlsbad, CA, USA). Both miR-99a and U6 RNA were assayed in duplicate in the reaction plate. Real-time qRT-PCR was performed on an Applied Biosystems 7900HT real-time qRT-PCR instrument. We performed PCR for 15 s at 95°C and 1 min at 60°C for 40 cycles followed by thermal denaturation. The expression of miR-99a relative to U6 RNA (control) was determined using the 2^−ΔCT^ method [41]. To simplify the presentation of the data, the relative expression values were multiplied by 10^2^.

### Development of Glioblastoma Xenograft Models

Our animal studies were conducted in strict accordance with the recommendations in the ‘Guide for the Care and Use of Laboratory Animals’ of the National Institutes of Health and also approved by the Institutional Animal Care and Use Committee (IACUC) of University of South Carolina (Columbia, SC, USA). All surgical procedures were performed under anesthesia condition and all efforts were made to minimize suffering to the animals. For using in this investigation, six-week-old female athymic (nu/nu) mice were purchased from Charles River Laboratories (Wilmington, MA, USA). The mice were housed in sterilized filter-topped cages and maintained in a pathogen-free animal facility. Mice were injected subcutaneously with human glioblastoma U87MG or U118MG cells (5×10^6^) in 100 µl of a (1∶1) mixture of serum-free 1×RPMI 1640 and Matrigel (BD Biosciences, CA, USA) for development of xenografts, as we described previously [42]. Tumors reached around 10 mm in diameter after 10 days and then treatments were carried out.

### Administration of Therapeutic Agents

The tumor-bearing mice were categorized into five separate experimental groups (control, anti-miR-99a, miR-99a, photofrin, and photofrin+miR-99a). We employed 6 animals per treatment group. Mice bearing U87MG or U118MG tumor were injected via tail vein with photofrin (10 mg/kg) after 10^th^ day of tumor xenotransplantation. After 24 h, animals were anesthetized with intraperitoneal injection of ketamine (100 mg/kg) and tumors were subjected to radiation under sterile conditions. Quantum Warp 10 (Quantum Devices, Barneveld, WI, USA), which generated near infra red light of 670 nm, was used for the radiation therapy with fluencies of 100 J/cm^2^ at a fluency rate of 50 mW/cm^2^ [43]. Animals in control group were kept in the dark without any treatment for the entire duration of the experiment. An equal volume of either anti-miR-99a or miR-99a and atelocollagen [44] (0.1% in PBS, pH 7.4) were mixed for 1 h at 4°C. Finally, anti-miR-99a or miR-99a (50 µg) with 0.05% atelocollagen in 200 µl was injected (via tail vein) into each mouse on 14^th^, 17^th^, and 20^th^ days.

### Histopathological Changes after Photofrin Based PDT and miR-99a Treatments

The animals were treated for a total of 11 days (as described above) and sacrificed under isoflurane anesthesia on 21^st^ day of tumor inoculation and then tumors were surgically removed. We took the weight of each tumor to assess its regression due to a treatment. Each tumor was then divided into two halves. One half of the tumor was quickly frozen in liquid nitrogen and stored at −80°C. The other half of the tumor was quickly frozen (−70°C) in Optima Cutting Temperature (OCT) media (Fisher Scientific, Suwanee, GA, USA) for cutting 5 µm sections of tumor in OCT with Microm HM 505N Cryostat Microtome Freezing (Labequip, Markham, Ontario, Canada). Then, histopathological changes in the tumor sections were examined under the light microscopy after conventional hematoxylin and eosin (H&E) staining [45].

### Antibodies and Western Blotting

After photofrin based PDT or/and miR-99a administration as described above, protein samples were isolated from the gliobalstoma U87MG and U118MG cells and xenografts. Protein samples were resolved by dodecyl sulfate-polyacrylamide gel electrophoresis (SDS-PAGE). Gels from all SDS-PAGE experiments were analyzed by Western blotting using the primary IgG antibody against VEGF, basic fibroblast growth factor (b-FGF), epidermal growth factor receptor (EGFR), matrix metalloproteinase-2 (MMP-2), MMP-9, p-Akt, nuclear factor-kappa B (NF-κB), phosphatase and tensin homolog deleted on chromosome 10 (PTEN), FGFR3, phosphatidylinositol-3-kinase (PI3K), Akt, p53, caspase-9, caspase-3, or β-actin (as a loading control). All primary IgG antibodies were procured from Santa Cruz Biotechnology (Santa Cruz, CA, USA). The horseradish peroxidase conjugated goat anti-mouse or anti-rabbit secondary IgG antibody (ICN Biomedicals, Aurora, OH, USA) was used for detection of the primary IgG antibodies. Western blots were incubated with enhanced chemiluminescence (ECL) detection reagents (Amersham Pharmacia, Buckinghamshire, UK) and exposed to X-OMAT AR films (Eastman Kodak, Rochester, NY, USA) for autoradiography. The autoradiograms were scanned on an EPSON Scanner using Photoshop software (Adobe Systems, Seattle, WA, USA).

### Statistical Analysis

The results from some of the experiments were analyzed for statistical significance using Minitab® 16 statistical software (Minitab, State College, PA, USA). Data were expressed as mean ± standard error of mean (SEM) of separate experiments (*n*≥3) and compared by one-way analysis of variance (ANOVA) followed by the Fisher’s post-hoc test. Difference between control (CTL, the untreated group) and a treatment was considered significant at *P*<0.05.

## Results

### Comparison of Photofrin Uptake by Different Human Glioblastoma Cell Lines

**Figure 1:**
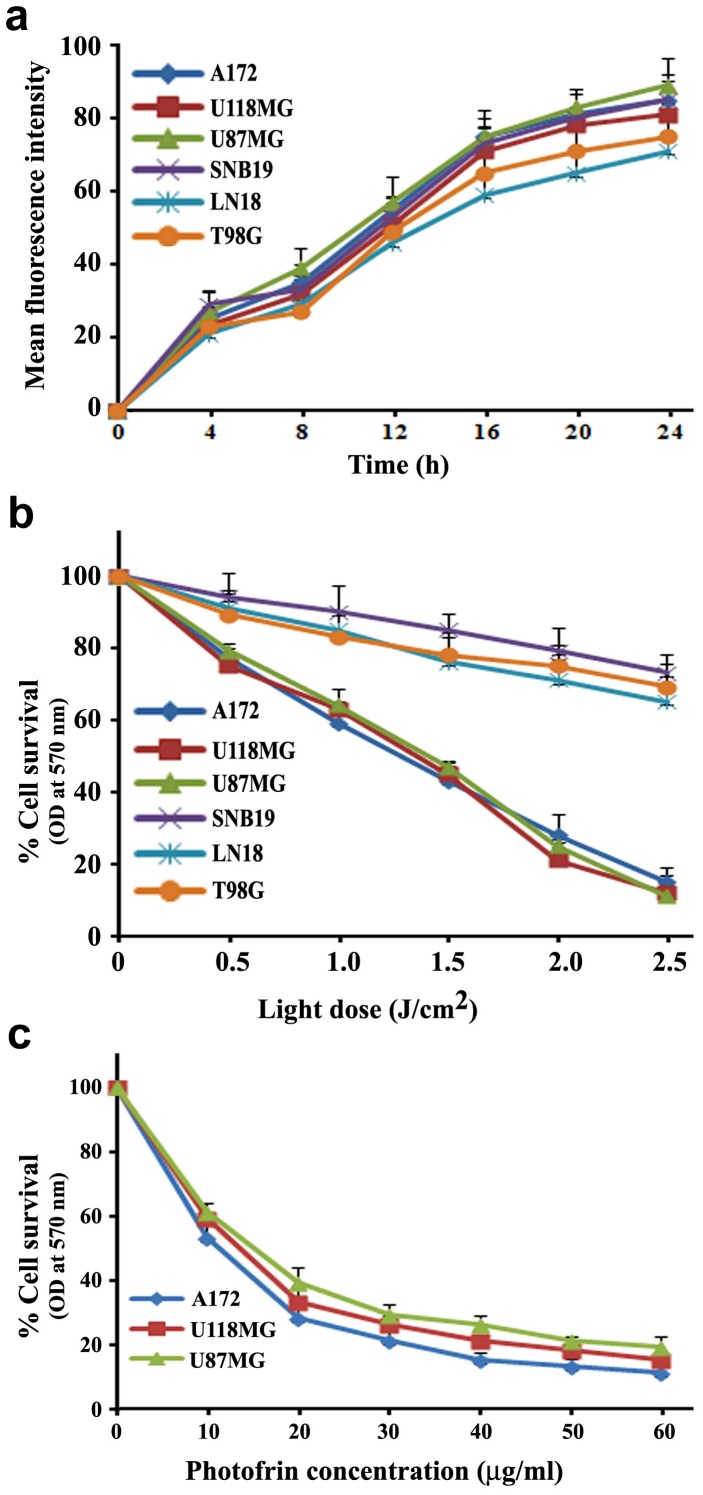
Uptake and cytotoxicity of photofrin in human glioblastoma cells. Human glioblastoma A172, U118MG, U87MG, SNB19, LN18, and T98G cells were incubated with 1 µg/ml **photofrin.** (a) Uptake of photofrin was time-dependent. (b) Photocytotoxic effect of photofrin was 670 nm light dose-dependent. (c) Photocytotoxic effect was photofrin dose-dependent. Data are presented as mean ± SEM of three independent experiments.

Optimum uptake of photofrin by all six glioblastoma cell lines (p53 wild-type and p53 mutant) was investigated before irradiation (Fig. 1a). The uptake kinetics was determined by measuring the cellular fluorescence signal using a flow cytometer with a 610-nm long pass filter. The results were expressed as mean fluorescence signal per cell. The fluorescence signal generated from photofrin elevated gradually reaching a maximum at around 24 h in all cell lines.

### Assessment of Photocytotoxic Effects on Different Human Glioblastoma Cell Lines

After determining optimum incubation times for photofrin, all six glioblastoma cell lines were subjected to photofrin based PDT with light doses ranging from 0 to 2.5 J/cm^2^ with 0.5 J/cm^2^ increment. After 24 h incubation, residual cell viability was determined by the MTT assay (Fig. 1b). Significant cell death was observed in p53 wild-type glioblastoma cell lines (A172, U87MG, and U118MG) with the use of as low as 0.5 J/cm^2^ light dose and the cytotoxicity was increased with the increase in light dose. Although p53 mutant glioblastoma cell lines (SNB19, LN18, and T98G) did not respond effectively to photofrin based PDT, all p53 wild-type glioblastoma cell lines (A172, U87MG, and U118MG) displayed almost similar growth inhibition and decrease in cell viability after PDT with photofrin doses ranging from 0 to 60 µg/ml (Fig. 1c).

### Induction of Morphological and Biochemical Features of Apoptosis after Photofrin Based PDT

**Figure 2:**
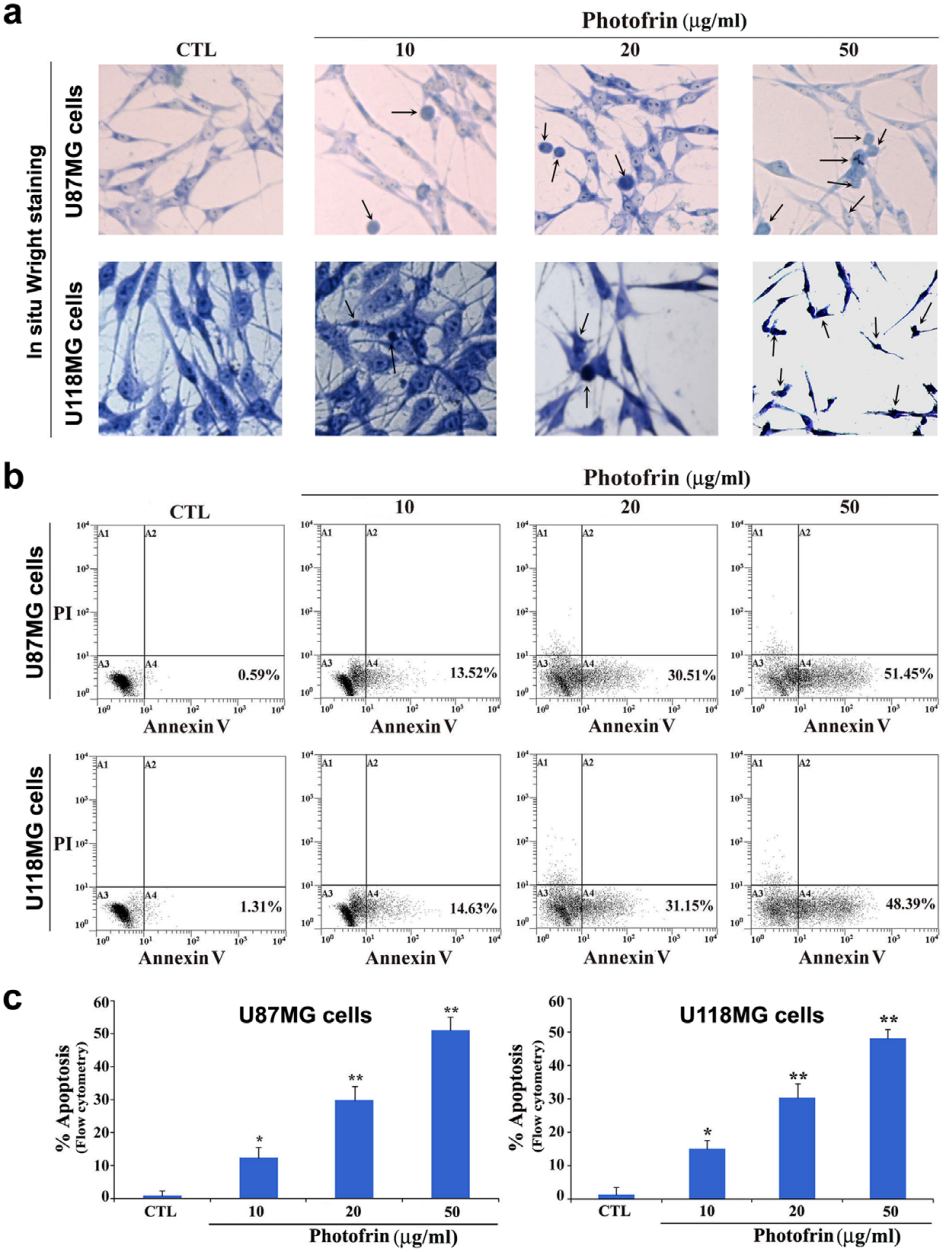
Determination of induction of morphological and biochemical features of apoptosis in human glioblastoma cells following photofrin based PDT. Treatments: control (CTL), 10, 20, and 50 µg/ml photofrin incubation for 4 h followed by irradiation with 670 nm light dose of 1 J/cm^2^. (a) In situ Wright staining to examine morphological features of apoptosis. (b) Annexin V-FITC/PI double staining and flow cytometric analysis of apoptotic populations after the treatments. Photofrin based PDT induced significant population of cells in A4 area, indicating induction of a biochemical feature of apoptotic death. (c) Determination of percentages of apoptosis based on biochemical feature revealed by Annexin V-FITC staining. Significant difference between CTL and a treatment was indicated by *P<0.05 or **P<0.01.

The morphological and biochemical features of apoptosis were examined using the in situ Wright staining and flow cytometry of Annexin V-FITC/PI stained cells, respectively (Fig. 2). In situ Wright staining showed the morphological features of apoptosis after photofrin dose-dependent PDT in U87MG and U118MG cell lines (Fig. 2a). Characteristic morphological features of apoptotic cells included shrinkage of cell volume, chromatin condensation, and membrane-bound apoptotic bodies that appeared prominently following treatment of cells with photofrin (50 µg/ml) followed by irradiation with light dose of 1 J/cm^2^ (Fig. 2a). An increase in population of Annexin V-FITC positive and PI negative cells in A4 area indicated occurrence of apoptosis, as shown in both U87MG and U118MG cell lines following PDT with different doses of photofrin (Fig. 2b). Based on flow cytometric analysis of Annexin V-FITC positive cells, we determined the percentages of apoptosis in U87MG and U118MG cell lines (Fig. 2c). Compared with control cells, PDT with 50 µg/ml photofrin very significantly increased the percentages of the Annexin V-FITC positive populations in both cell lines (Fig. 2a). The increase in Annexin V-FITC positive cells after the photofrin based PDT indicated a prominent biochemical feature of apoptosis in glioblastoma cells. Flow cytometry data obtained from three independent experiments were used to determine the amounts of apoptosis in both cell lines (Fig. 2c).

### Photofrin Based PDT Prevented Invasion of Human Glioblastoma Cells

**Figure 3:**
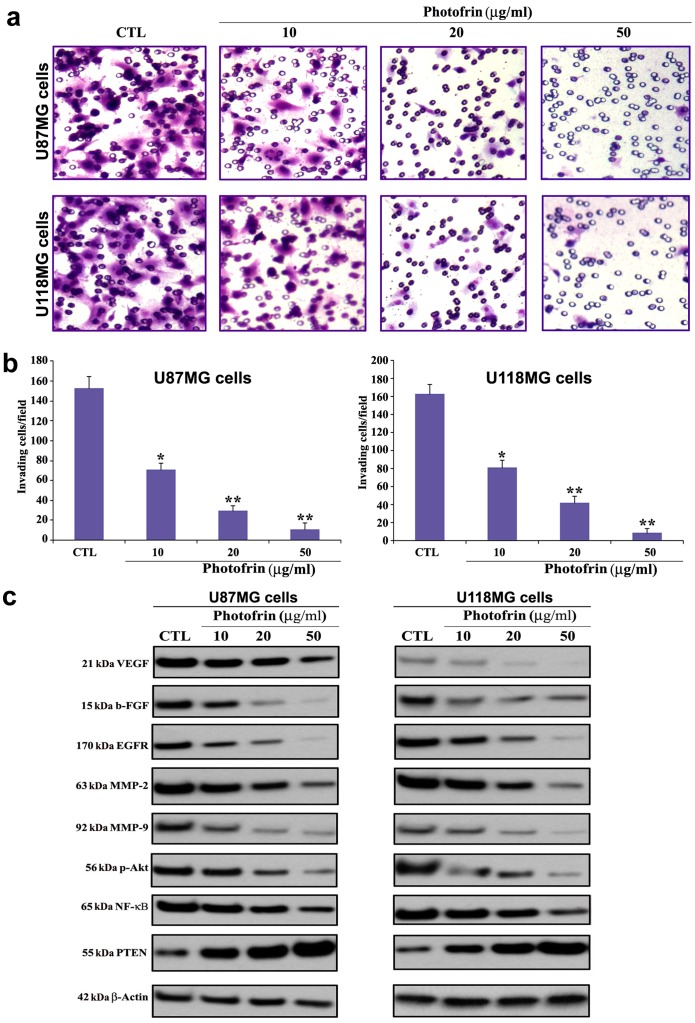
Changes in capability of cell invasion and alterations in expression of angiogenic, invasive, and survival factors in glioblastoma cells after photofrin based PDT. Treatments: control (CTL), 10, 20, and 50 µg/ml photofrin incubation for 4 h followed by irradiation with 670 nm light dose of 1 J/cm^2^. (a) Representative Matrigel invasion assay (48 h) using U87MG and U118MG cells. A significant reduction in the number of invaded cells indicated the decrease in invasive capability of the cells after dose-dependent photofrin based PDT. (b) Quantitative evaluation of Matrigel invasion. Data indicate mean ± SEM of 10 randomly selected microscopic fields from 3 independent wells. Significant difference between CTL and a treatment was indicated by *P<0.05 or **P<0.01. (c) Western blotting using the primary IgG antibodies against VEGF, b-FGF, EGFR, MMP-2, MMP-9, p-Akt, NF-κB, and PTEN, and β-actin (loading control).

Invasion ability is indicated by the number of cells that migrate through the reconstituted Matrigel layer to the bottom surface of the porous membrane. PDT with 10 µg/ml photofrin caused a significant reduction in invasiveness of both cell lines **(** **Fig. 3a** **)**. At higher concentration of photofrin (50 µg/ml) further reduction in invasion of glioblastoma cells was observed where less than 10% cells invaded through the Matrigel layer (Fig. 3b). We examined the alterations in expression of angiogenic factors, growth factors, and invasive factors by Western blotting following photofrin based PDT (Fig. 3c). Photofrin based PDT could suppress the expression of angiogenic factors (VEGF and b-FGF), a growth factor receptor (EGFR), and invasive factors (MMP-2 and MMP-9) in U87MG and U118MG cells (Fig. 3c). Photofrin based PDT could also effectively suppress activation (phosphorylation) of Akt and NF-κB survival pathways. One of the salient observations made in the present study was the incremental expression of the tumor suppressor PTEN in response to photofrin based PDT in both U87MG and U118MG cells. We found that increase in expression of PTEN was associated with suppression of expression of angiogenic and growth factors. Thus, photofrin based PDT was highly efficient in down regulating angiogenic factors, survival pathways, and upregulating the tumor suppressor PTEN in human glioblastoma U87MG and U118MG cells.

### Photofrin Based PDT Inhibited in vitro Angiogenic Network Formation and Expression of VEGF

**Figure 4:**
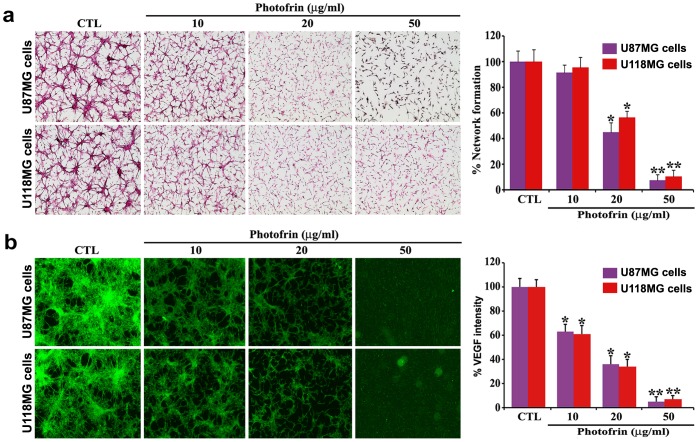
Association of network formation ability of HME cells with VEGF expression in gliblastoma U87MG or U118MG cells in co-cultures. U87MG and U118MG cells were grown separately on chamber slides and treated with different doses of photofrin. Treatments: control (CTL), 10, 20, and 50 µg/ml photofrin incubation for 4 h followed by irradiation with 670 nm light dose of 1 J/cm^2^. After 24 h, HME cells were co-cultured with glioblastoma cells. Quantitative data are shown as means ± SEM of six independent experiments in each group. Significant difference between CTL and a treatment was indicated by *P<0.05 or **P<0.01. (a) Effect of photofrin based PDT on network formation ability of HME cells in co-cultures. The co-cultures were terminated at 72 h and immunohistochemically stained for expression of the von Willebrand factor VIII in HME cells. Then, in vitro networks were quantified. (b) Effect of photofrin based PDT on VEGF expression in glioblastoma cells in co-cultures. Following the same treatments and incubations as described above, another set of same network formation experiments were conducted for in situ immunofluorescence microscopic studies using the FITC conjugated anti-VEGF antibody to determine the levels of VEGF expression in glioblastoma cells in co-cultures.

We examined the in vitro angiogenic network formation in co-culture of HME cells and glioblastoma cells following photofrin based PDT (Fig. 4). The von Willebrand factor VIII is a characteristic marker of the endothelial cells. Synthesis and secretion of VEGF from glioblastoma cells can support HME cells for angiogenic network formation. The co-culture of HME cells and U87MG or U118MG cells showed capillary-like network formation ability of HME cells (Fig. 4a). Such network formation ability of HME cells was significantly reduced in co-culture with glioblastoma cells from photofrin dose-dependent PDT (Fig. 4a). Network formation was almost completely inhibited after 50 µg/ml photofrin treatment followed by radiation therapy (1 J/cm^2^). Quantitative evaluation of in vitro network formation ability of HME cells showed that PDT with 10, 20, and 50 µg/ml photofrin doses caused 9, 55, and 93% inhibition of network formation, respectively, with U87MG cells, whereas 5, 44, and 90% inhibition of network formation, respectively, with U118MG cells (Fig. 4a). We performed another set of same angiogenic network formation experiments for our in situ immunofluorescence microscopic studies using the FITC conjugated anti-VEGF antibody to examine any changes in expression of VEGF in glioblastoma U87MG and U118MG cells due to dose-dependent photofrin based PDT (Fig. 4b). Quantitative analyses of immunofluorescence intensities showed that expression of VEGF was almost completely (95%) inhibited in both glioblastoma U87MG and U118MG cell lines after photofrin (50 µg/ml) based PDT (1 J/cm^2^). Decrease in expression of VEGF in glioblastoma cells was correlated with the decease in network formation ability of the HME cells in co-cultures.

### Photofrin Based PDT Induced Apoptosis with DNA Fragmentation and Laddering

**Figure 5:**
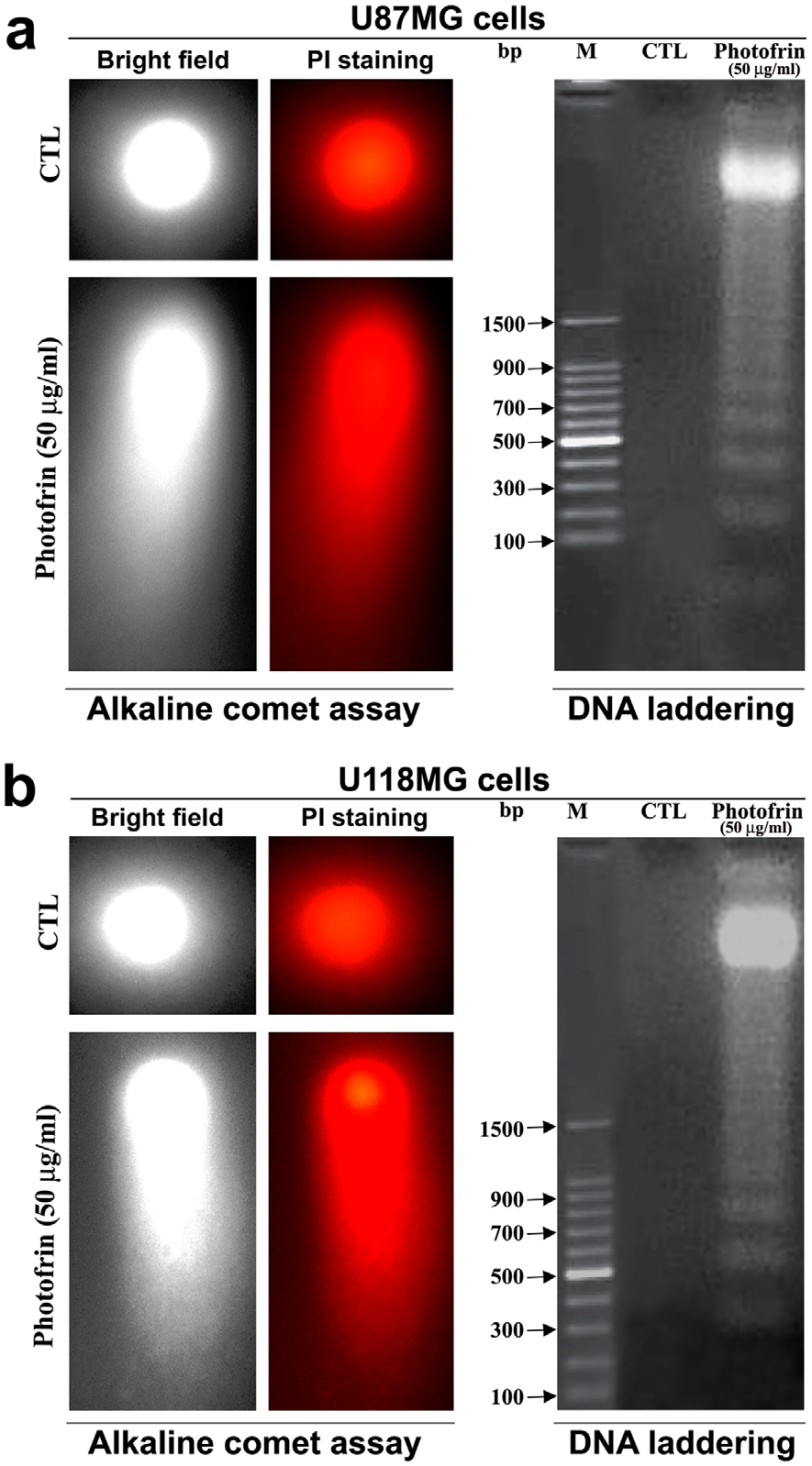
Alkaline comet assay and agarose gel electrophoresis to examine DNA fragmentation patterns in U87MG and U118MG cells after photofrin based PDT. (a) Photomicrographs showing the DNA fragmentation patterns in U87MG cells. Two U87MG cells, one control (CTL) and another cell with highly damaged DNA due to photofrin based PDT, are shown in alkaline comet assay. Also, cells were treated with 50 µg/ml photofrin and irradiated with 670 nm light (1 J/cm^2^) and incubated for 3 h before isolation of total genomic DNA for DNA laddering assay. The CTL showed intact DNA whereas DNA ladder appeared due to photofrin based PDT. (b) Photomicrographs showing the DNA fragmentation patterns in U118MG cells. Two U118MG cells, one CTL and another cell with highly damaged DNA due to photofrin based PDT, are shown in alkaline comet assay. Also, cells were treated with 50 µg/ml photofrin and irradiated with 670 nm light (1 J/cm^2^) and incubated for 3 h before isolation of total genomic DNA for DNA laddering assay. The CTL showed intact DNA whereas DNA ladder appeared due to photofrin based PDT.

The influence of photofrin based PDT on the mode of cell death was first determined by the alkaline comet assay. A characteristic distribution of comets was noticed at 3 h after PDT with photofrin (50 µg/ml) in both U87MG and U118MG cell lines (Fig. 5). Single-strand DNA fragmentation in alkaline comet assay indicated a possibility of induction of apoptotic cell death. This apoptotic program was most likely triggered in the cells to cause DNA double-strand breaks as well. The most apoptotic nuclei were noted after 2 h in both cells and at the highest dose of photofrin based PDT. The formation of DNA ladder, an indicator of double-strand breaks and a hallmark of apoptosis**,** was also observed after photofrin (50 µg/ml) based PDT in U87MG cells (Fig. 5a) as well as in U118MG cells (Fig. 5b). No evidence of DNA ladder was found in the untreated control cells.

### Photofrin Based PDT followed by Ectopic Overexpression of miR-99a Increased Apoptotic Death

**Figure 6:**
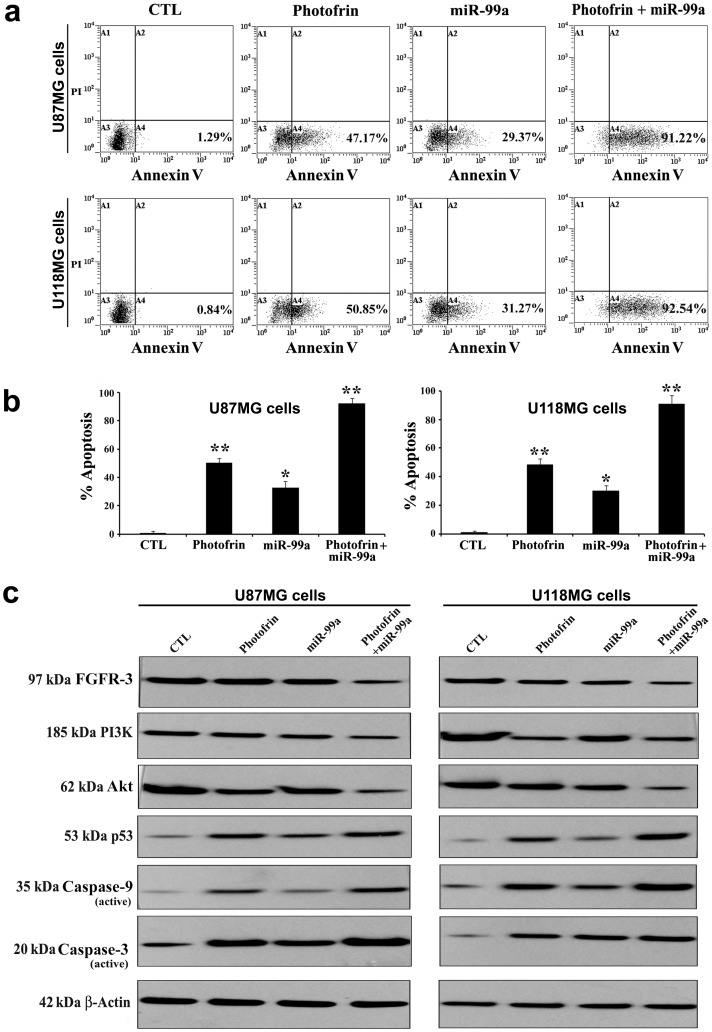
Augmentation of efficacy of photofrin based PDT by miR-99a overexpression for induction of apoptosis in U87MG and U118MG cells. Cells were seeded into 6-well plates at 5×10^5^ cells per well in triplicate. After 24 h, cells were treated with 50 µg/ml photofrin and irradiated with 670 nm light of 1 J/cm^2^. Following 4 h incubation, cells were transfected with 50 nM pre-miR-99a mimic and incubated for another 24 h. (a) Cells were collected for estimation of apoptosis by Annexin V-FITC/PI double staining and flow cytometry. (b) Percentages of apoptotic cells from three independent experiments were shown in bar diagrams. Significant difference between control (CTL) and a treatment was indicated by *P<0.05 or **P<0.01. (c) Representative Western blots (*n* ≥3) showed expression of 97 kDa FGFR3, 185 kDa PI3K, 62 kDa Akt, 53 kDa p53, 35 kDa caspase-9 (active), 20 kDa caspase-3 (active), and 42 kD β-actin.

The efficacy of combination of photofrin based PDT and miR-99a overexpression in increasing apoptosis in human glioblastoma U87MG and U118MG cell lines was analyzed by flow cytometry and Western blotting (Fig. 6). As observed from flow cytometric analyses, photofrin based PDT alone could induce some apoptosis, which appeared to be significantly increased with combination of photofrin based PDT and miR-99a transfection (Fig. 6a, 6b). To determine the possible signaling mechanisms for anti-proliferative and pro-apoptotic activity of photofrin based PDT and miR-99a overexpression, we performed Western blotting and detected alterations in the expression of the regulatory factors for cell growth and apoptosis in both cell lines (Fig. 6c). Photofrin based PDT and miR-99a overexpression very efficiently suppressed the levels of FGFR3, PI3K, and Akt to promote p53-mediated mitochondrial caspase-dependent apoptosis. Apoptosis indeed occurred with the activation of caspase-9 and caspase-3.

### Quantification of miR-99a Expression after Photofrin Based PDT or/and miR-99a Transfection

**Figure 7:**
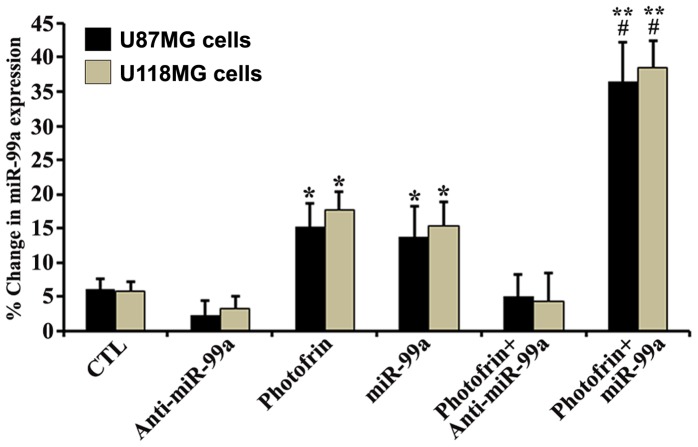
Real-time qRT-PCR analyses of miR-99a expression in U87MG and U118MG cells after photofrin based PDT and miR transfection. Cells were seeded, incubated, treated with photofrin, and irradiated with 670 nm light dose of 1 J/cm^2^. After 4 h incubation, cells were transfected with 50 nM anti-miR-99a mimic or miR-99a mimic and incubated for another 24 h. Total RNA was extracted and cDNA was synthesized using gene specific primers, and real-time qRT-PCR analysis was performed for relative expression of miR-99a after normalizing with expression of U6 RNA (control) in glioblastoma U87MG and U118MG cells. Significant difference between untreated control (CTL) and photofrin based PDT or miR-99a transfection was indicated by *P<0.05 or **P<0.01. Significant difference between a single therapy and combination therapy was indicated by ^#^ *P*<0.05.

We have performed real-time qRT-PCR to examine the levels of expression of the tumor suppressor miR-99a in photofrin treated glioblastoma U87MG and U118MG cells after irradiation, without or with miR-99a transfection (Fig. 7). We also kept appropriate control (no photofrin and irradiation) cells and anti-miR-99a mimic and miR-99a mimic transfected cells for relative efficacy studies. Upregulation of miR-99a occurred to some extent in both cell lines following photofrin based PDT when compared with the untreated control cells, but upregulation of miR-99a reached a maximum level in the cells that were subjected to combination of photofrin based PDT and miR-99a transfection. Anti-miR-99a transfection inhibited the photofrin based PDT enhancement of miR-99a expression in both glioblastoma cell lines.

### Efficacy of Photofrin Based PDT and miR-99a Overexpression for Tumor Regression

**Figure 8:**
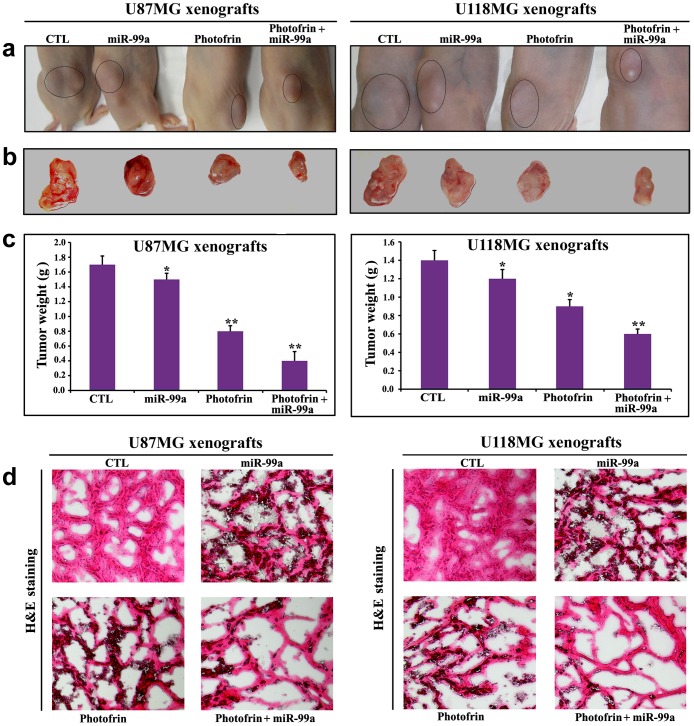
Regression of U87MG and U118MG tumors in nude mice and histopathological changes in tumor sections. (a) Nude mice with U87MG and U118MG xenografts, (b) representative tumors removed surgically, (c) determination of tumor weight, and (d) evaluation of histopathological changes after the treatments. Mice with xenografts were treated for 11 days. Treatments: control (CTL) did not receive any treatment but tumor bearing mice (10^th^ day after tumor implantation) were injected with photofrin (10 mg/kg) by tail vein, and 24 h later, 670 nm light was delivered to the tumor with fluencies of 100 J/cm^2^ at a fluency rate of 50 mW/cm^2^. We used 100 J/cm^2^ to expose most of the tumor cells to radiation (32). Then, the mixture of miR-99a mimic (50 µg) and 0.05% atelocollagen in 200 µl was injected (via tail vein) into each mouse on 14^th^, 17^th^, and 20^th^ days. All animals were sacrificed on 21^st^ day. We used 6 animals per group. Significant difference between CTL group and a treatment group was indicated by *P<0.05 or **P<0.01.

Further, our in vivo studies showed that combination of photofrin based PDT and miR-99a overexpression very effectively reduced the growth of both U87MG and U118MG xenografts in athymic nude mice (Fig. 8a, 8b, 8c). Compared with untreated control or a monotherapy, combination therapy showed significant reductions in tumor weight. Following treatments, H&E staining of tumor sections showed that control tumors maintained characteristic growth, photofrin based PDT or miR-99a overexpression alone induced cell death to some extent, but combination of photofrin based PDT and miR-99a overexpression dramatically increased cell death in both U87MG and U118MG xenograft models (Fig. 8d).

### Down Regulation of PI3K/Akt Signaling Pathway Promoted p53-mediated Mitochondrial Caspase-dependent Apoptosis in Glioblastoma Xenografts

**Figure 9:**
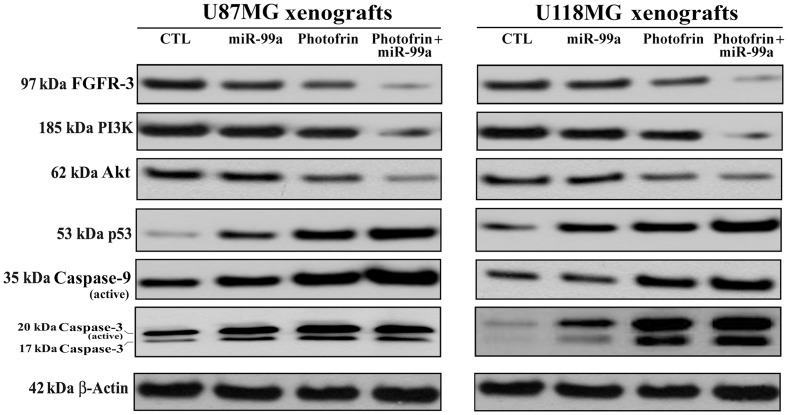
Changes in expression of survival and apoptotic proteins in U87MG and U118MG xenografts. Mice with xenografts were treated for 11 days. Treatments: control (CTL) did not receive any treatment but tumor bearing mice (10^th^ day after tumor implantation) were injected with photofrin (10 mg/kg) by tail vein, and 24 h later, 670 nm light was delivered to the tumor with fluencies of 100 J/cm^2^ at a fluency rate of 50 mW/cm^2^. We used 100 J/cm^2^ to expose most of the tumor cells to radiation (32). Then, the mixture of miR-99a mimic (50 µg) and 0.05% atelocollagen in 200 µl was injected (via tail vein) into each mouse on 14^th^, 17^th^, and 20^th^ days. All animals were sacrificed on 21^st^ day. Representative Western blots (*n*≥3) showed expression of 97 kDa FGFR-3, 185 kDa PI3K, 62 kDa Akt, 53 kDa p53, 35 kDa caspase-9 (active), 20 kDa caspase-3 (active), and 42 kD β-actin.

Western blotting showed the down regulation of FGFR3, PI3K, and Akt but upregulation of p53, active caspase-9, and active caspase-3 following combination therapy in both glioblastoma U87MG and U118MG xenograft models (Fig. 9). So, our results from xenograft models further confirmed that combination of photofrin based PDT and miR-99a transfection inhibited FGFR3 and PI3K/Akt signaling pathways to promote p53-mediated mitochondrial caspase-dependent apoptosis in human glioblastomas in vivo.

## Discussion

The most important outcome of this investigation is the establishment of molecular basis of the efficacy of the combination of photofrin based PDT and miR-99a overexpression in controlling the growth of human glioblastoma cells (p53 wild-type) in**vitro and in vivo. We first examined the uptake kinetics of photofrin in both p53 wild-type and p53 mutant glioblastoma cell lines. The tumor suppressor protein p53 co-ordinates DNA repair, cell cycle progression, and DNA damage to determine if induction of apoptosis is necessary [46]. As a transcription factor, p53 regulates at least 100 genes participating in these processes [47]. p53 plays a central role in the cellular response to oxidative stress [48]. This can be the reason that only p53 wild-type glioblastoma cells are greatly responsive to photofrin based PDT. Although photofrin uptake was observed to be almost same by all glioblastoma cell lines that we examined, cell viability in p53 wild-type cells clearly decreased depending on photofrin and light doses. Following treatments, cellular responses including inhibition of proliferation, induction of apoptosis, cell invasion, angiogenesis, and DNA fragmentation were also investigated. The increases in apoptosis following photofrin based PDT in two p53 wild-type glioblastoma cell lines was confirmed by both in situ Wright staining and Annexin V-FITC binding assay.

Photofrin induces cell cycle arrest at G0/G1 phase, which may be an explanation for its role in induction of apoptotic death [49]. The tumor suppressor PTEN negatively regulates PI3K/Akt pathway by dephosphorylating phosphatidylinositol-3,4,5-triphosphate [50]. We found that photofrin based PDT could upregulate PTEN to inhibit growth factor mediated angiogenic, invasive, and survival pathways (VEGF, b-FGF, EGFR, MMP-2, MMP-9, p-Akt, and NF-κB). Photofrin at a higher concentration effectively blocked the survival advantages in two different p53 wild-type glioblastoma U87MG and U118MG cell lines leading to induction of apoptosis, suggesting that photofrin based PDT could be a novel strategy for controlling the growth of p53 wild-type glioblastoma cells. The efficacy of photofrin based PDT also inhibited production of the angiogenic factor VEGF by U87MG and U118MG cells to prevent network formation ability of HME cells in co-cultures.

Alkaline comet assay and also neutral gel electrophoresis assay proved the occurrence DNA fragmentation and DNA laddering, respectively, due to induction of apoptotic death in human glioblastoma cells at high dose of photofrin based PDT. A recent study showed that the upregulation of the miR-99 family following radiation decreased the efficiency of repair factor recruitment and the rate of DNA repair after a second exposure to radiation [35]. The induction of miR-99a expression represents a switch by which cells subjected to multiple rounds of radiation are directed away from continuing to repair their DNA.

We observed increases in expression of miR-99a in glioblastoma cells after photofrin based PDT. These results clearly suggested that miR-99a could act as a tumor suppressor and an increase in level of expression of miR-99a could further increase amounts of apoptosis in glioblastoma cell lines. We studied the effects of miR-99a overexpression that enhanced the efficacy of photofrin based PDT for induction of apoptosis in glioblastoma cell lines. We observed induction of more apoptosis when miR-99a transfection was carried out using Lipofectamine after irradiation. Most gene therapy vehicles are taken into the cells by endocytosis. Poor endosomal release inhibits gene transfer. Photofrin is localized specifically in the membranes of endocytic vesicles and, following activation by light, it induces the rupture of the vesicular membranes. This photochemical reaction may help the miR-99a transfection process.

Survival signals induced by FGFR3 are mediated mainly via PI3K/Akt pathway, which may play a major role in drug resistance and decrease in radio sensitivity [51]. PI3K/Akt pathway activation is related to tumor cell resistance to both chemotherapy and radiation [52]. Here, we investigated whether miR-99a overexpression could significantly enhance the therapeutic efficacy of photofrin based PDT. An earlier research has shown that activation of PI3K/Akt pathway, evaluated by measuring Akt phosphorylation, may be a marker in predicting the response to radiation therapy in head-and-neck cancer [53].

It is important to note that PI3K/Akt pathway is a key regulator of cell growth and survival in many cancers, including FGFR3 regulated cancers [54]. Here, combination of photofrin based PDT and miR-99a overexpression very effectively down regulated expression of FGFR3, which might directly or indirectly inhibit expression of PI3K and Akt, with an increase in expression of p53. Notably, p53 plays crucial role in regulating cell cycle progression and functions as a tumor suppressor. It is known that p53 can induce apoptosis through mitochondrial caspase-dependent pathway. Our results showed that an elevation in level of p53 after combination therapy induced the mitochondrial pathway of apoptosis for activation of caspase-9, which in turn activated caspase-3 for completion of apoptosis in glioblastoma cells both in culture and xenograft models.

In conclusion, photofrin based PDT can be a very useful adjuvant therapy in the treatment of human p53 wild-type glioblastomas because of its impressive ability to reduce not only tumor cell proliferation but also tumor cell invasion. The results of this study revealed the molecular basis for the effectiveness of combination of photofrin based PDT and miR-99a transfection for inhibiting growth of the p53 wild-type glioblastoma cells in vitro and in vivo.
